# Outcome of *Enterococcus faecalis* infective endocarditis according to the length of antibiotic therapy: Preliminary data from a cohort of 78 patients

**DOI:** 10.1371/journal.pone.0192387

**Published:** 2018-02-20

**Authors:** Juan M. Pericàs, Carlos Cervera, Asunción Moreno, Cristina Garcia-de-la-Mària, Manel Almela, Carles Falces, Eduard Quintana, Bàrbara Vidal, Jaume Llopis, David Fuster, Carlos A. Mestres, Francesc Marco, Jose M. Miró, on behalf of the Hospital Clinic Endocarditis Study Group

**Affiliations:** 1 Infectious Diseases Service, Hospital Clínic, Institut d’Investigacions Biomèdiques August Pi i Sunyer, University of Barcelona, Barcelona, Spain; 2 Division of Infectious Diseases, University of Alberta, Edmonton, Canada; 3 Microbiology Service, Hospital Clínic, Institut d’Investigacions Biomèdiques August Pi i Sunyer, University of Barcelona, Barcelona, Spain; 4 Cardiology Service, Hospital Clínic, Institut d’Investigacions Biomèdiques August Pi i Sunyer, University of Barcelona, Barcelona, Spain; 5 Cardiovascular Surgery Service, Hospital Clínic, Institut d’Investigacions Biomèdiques August Pi i Sunyer, University of Barcelona, Barcelona, Spain; 6 Department of Statistics, Faculty of Biology, University of Barcelona, Barcelona, Spain; 7 Nuclear Medicine Service, Hospital Clínic, Institut d’Investigacions Biomèdiques August Pi i Sunyer, University of Barcelona, Barcelona, Spain; 8 Department of Cardiovascular Surgery, UniversitätsSpital Zürich, Zurich, Switzerland; 9 ISGlobal, Microbiology Service, Hospital Clínic, University of Barcelona, Barcelona, Spain; Azienda Ospedaliera Universitaria di Perugia, ITALY

## Abstract

**Background:**

International guidelines recommend 4 weeks of treatment with ampicillin plus gentamicin (A+G) for uncomplicated native valve *Enterococcus faecalis* infective endocarditis (EFIE) and 6 weeks in the remaining cases. Ampicillin plus ceftriaxone (A+C) is always recommended for at least 6w, with no available studies assessing its suitability for 4w. We aimed to investigate differences in the outcome of EFIE according to the duration (4 versus 6 weeks) of antibiotic treatment (A+G or A+C).

**Methods:**

Retrospective analysis from a prospectively collected cohort of 78 EFIE patients treated with either A+G or A+C.

**Results:**

32 cases (41%) were treated with A+G (9 for 4w, 28%) and 46 (59%) with A+C (14 for 4w, 30%). No significant differences were found in 1-year mortality according to the type of treatment (31% and 24% in A+G and A+C, respectively; P = 0.646) or duration (26% and 27% at 4 and 6w, respectively; P = 0.863). Relapses were more frequent among survivors treated for 4w than in those treated for 6w (3/18 [17%] at 4w and 1/41 [2%] at 6w; P = 0.045). Three out of 4 (75%) relapses occurred in cirrhotic patients.

**Conclusions:**

A 4-week course of antibiotic treatment might not be suitable neither for A+G nor A+C for treating uncomplicated native valve EFIE.

## Introduction

Enterococci are the third most common causal agent of infective endocarditis (IE) worldwide [1] and are becoming increasingly prevalent [2]. The combination of beta-lactams and aminoglycosides, mainly ampicillin/gentamicin, has been the treatment of choice from the 1950s until the publication of the latest American Heart Association (AHA) and European Society of Cardiology (ESC) guidelines [3–7]. The decision to initiate long-course treatment (6 weeks) or short-course treatment (4 weeks) of ampicillin/gentamicin has traditionally been based on the duration of symptoms (longer or shorter than 3 months) and the type of IE (native vs. prosthetic) [3–8].

Ampicillin/ceftriaxone is considered a good option for the treatment of *Enterococcus faecalis* IE (EFIE). Since the efficacy of double beta-lactam therapy was first described in the mid-1990s *in vitro* [9] and thereafter in animal models [10] and in clinical practice [10–13], this alternative has become increasingly used, especially in France and Spain. However, until the publication of the last European and American guidelines, ampicillin/ceftriaxone was only recommended as a second-line rescue option for high-level aminoglycoside-resistant (HLAR) strains and always using an 8-week course [3, 4]. In the latest guidelines of the AHA [5] and the ESC [6], ampicillin/ceftriaxone is always recommended for at least 6 weeks. The aim of this study was to compare the outcome of patients with EFIE treated with either ampicillin/gentamicin or ampicillin/ceftriaxone according to the duration of treatment (4 weeks vs 6 weeks) in order to address if the short-course treatment was adequate and whether a subgroup of patients could be identified to be safely treated for only 4 weeks.

## Methods

### Design

We performed a retrospective study of prospectively collected cases from a cohort attended in an 850-bed urban university hospital. All consecutive enterococcal IE episodes occurring between January 1997 and December 2013 were recorded in a specific database using a standardized case report form. The study population comprised patients with a definitive diagnosis of native or prosthetic valve infective endocarditis [14] caused by *E*. *faecalis* who were receiving treatment with ampicillin/gentamicin or ampicillin/ceftriaxone and whose antibiotic susceptibility test results were available. The Ethics Committee of the Hospital Clínic de Barcelona gave its approval to perform the study. All databases containing patients’ personal information were accordingly anonymized for storage and analysis.

All patients were initially treated with either ampicillin/gentamicin or ampicillin/ceftriaxone and aimed to complete 4 weeks or 6 weeks of treatment. The statistical analysis was performed on an intention-to-treat basis based on the initially scheduled length of treatment (see “Antibiotic treatment choice and characteristics”). All survivors had been followed for at least 1 year.

The demographic and clinical variables analyzed are displayed in Tables 1 and 2 and defined elsewhere [15]. Definitions of toxicity related to treatment and outcomes (relapses, in-hospital and 1-year mortality) were previously defined by our group [13].

**Table 1:** Demographic and baseline clinical characteristics of 78 patients with EFIE.

	A+G (N = 32)		A+C (N = 46)		*P*
	4 wk (N = 9)	6 wk (N = 23)	4 wk (N = 14)	6 wk (N = 32)	
Median age in years (IQR)	75 (69-76)	72 (68-78)	72 (65-80)	68 (63-76)	0.530
Male gender (%)	6 (67%)	18 (78%)	7 (50%)	19 (59%)	0.328
Transferred from another center	2 (22%)	12 (52%)	2 (14%)	5 (16%)	0.012
Median Charlson score (IQR)	2.0 (0-4)	3.0 (1-4)	3.0 (2-4)	2.0 (1-3)	0.805
Comorbidities					
Diabetes mellitus	3 (33%)	9 (39%)	3 (20%)	11 (36%)	0.734
Chronic renal failure	4 (44%)	4 (17%)	1 (7%)	11 (34%)	0.093
Hemodialysis	2 (22%)	1 (4%)	0	1 (3%)	0.087
Cancer	2 (22%)	6 (26%)	4 (29%)	5 (16%)	0.771
Chronic lung disease	1 (11%)	6 (26%)	2 (14%)	9 (28%)	0.630
Liver cirrhosis	1 (11%)	1 (4%)	3 (21%)	4 (13%)	0.253
Previous IE	0	1 (4%)	2 (14%)	8 (25%)	0.207
Type of acquisition					0.178
Community	3 (33%)	7 (30%)	5 (36%)	12 (38%)	
Nosocomial	5 (56%)	14 (61%)	3 (21%)	13 (41%)	
Non-nosocomial healthcare-associated	1 (11%)0	2 (9%)	6 (43%)	7 (22%)	

4-wk: 4-week course of antibiotic treatment; 6-wk: 6-week course of antibiotic treatment; A+C: ampicillin/ceftriaxone; A+G: ampicillin/gentamicin; IE: infective endocarditis; IQR: interquartile range

**Table 2:** Clinical profile and outcome of 78 patients with EFIE.

	A+G (N = 32)		A+C (N = 46)		*P*
	4 wk (N = 9)	6 wk (N = 23)	4 wk (N = 14)	6 wk (N = 32)	
Type of endocarditis					0.001
Native valve	9 (100%)	14 (61%)	14 (100%)	14 (44%)	
Prosthetic valve	0	9 (39%)	0	18 (56%)	
Duration of symptoms in days, median (IQR)	2 (2-15)	30 (11-60)	7 (3-15)	10 (3-30)	0.055
Echocardiographic features					
Presence of vegetations	7 (78%)	9 (87%)	20 (64%)	22 (69%)	0.373
Size of vegetations in mm, median (IQR)	10 (4-13)	9 (5-14)	7.5 (4.5-13)	10.5 (6-16)	0.713
Periannular complications	0	6 (26%)	0	4 (13%)	0.065
Clinical complications					
Heart failure (Killip ≥3)	0	10 (44%)	1 (7%)	14 (44%)	0.008
Renal failure	5 (56%)	15 (65%)	5 (36%)	10 (31%)	0.067
Major emboli	0	7 (30%)	0	13 (41%)	0.007
Persistent bacteremia	1 (11%)	1 (4%)	3 (21%)	1 (3%)	0.143
Adverse effects related to antibiotic treatment					
Vestibular toxicity and ototoxicity	2 (22%)	1 (4%)	0	0	0.023
Myelotoxicity	0	0	1 (7%)	0	0.295
Skin rash	0	1 (4%)	1 (7%)	1 (3%)	1.000
*C*. *difficile* diarrhea	0	0	0	2 (6%)	0.499
Superinfection due to betalactam-resistant agents	0	0	0	2 (6%)	0.499
Discontinuation of antibiotic therapy	2 (22%)	9 (39%)	1 (7%)	1 (3%)	0.003
Surgical treatment	2 (22%)	14 (61%)	3 (21%)	14 (44%)	0.062
Mortality					
In-hospital mortality	3 (33%)	6 (26%)	2 (14%)	8 (25%)	0.759
One-year mortality	3 (33%)	7 (30%)	3 (21%)	8 (25%)	0.901
Relapses*	1/6 (17%)	1/17 (6%)	2/12 (17%)	0/24 (0%)	0.170

4-wk: 4-week course of antibiotic treatment; 6-wk: 6-week course of antibiotic treatment; A+C: ampicillin/ceftriaxone; A+G: ampicillin/gentamicin; IE: infective endocarditis; IQR: interquartile range

* In patients surviving the first admission due to IE.

### Antibiotic treatment choice and characteristics

Following 1995 and 2005 AHA [3,7] and 2009 ESC [4] recommendations, ampicillin was administered at 2 g/4 h, and gentamicin at 3 mg/kg (up to 80 mg) in 3 doses as suggested by guidelines [3,4] or once daily based on the favorable results of a Danish study [16]. Gentamicin levels were monitored following 2005 AHA guidelines [3]. Ampicillin/ceftriaxone was administered as follows: ampicillin 2 g/4 h and ceftriaxone 2 g/12 h [11,13]. Duration of treatment (4 or 6 weeks) was based on AHA guidelines, which address duration of symptoms (more or less than 3 months), type of IE (native or prosthetic), and presence of complications (treatment was administered for 6 weeks in cases of major emboli, periannular abscess, intracardiac fistula, and new-onset severe heart failure) to recommend 4week course of ampicillin/gentamicin [3,5]. In our institution, ampicillin/ceftriaxone was preferably used above ampicillin/gentamicin, especially in elderly patients of those at risk to develop acute renal failure since the favorable results of Gavaldà et al. study [10], regardless the presence of HLAR. Furthermore, 4 weeks of ampicillin/ceftriaxone were indicated following the same recommendations as for ampicillin/gentamicin.

### Microbiological processing of samples

*E*. *faecalis* isolates were recovered from the 78 patients included in the study and frozen in skimmed milk at –80°C. Strains were identified using the API Rapid ID32 STREP device (bioMérieux, Marcy l’Etoile, France). HLAR was assessed using the E-test following the manufacturer’s recommendations (bioMérieux S.A., Marcy l’Etoile, France) and defined as a gentamicin minimum inhibitory concentration (MIC) >512 mg/L (HLGR) and/or a streptomycin MIC >1024 mg/L (HLSR). Following the guidelines of the CLSI [17] and the criteria defined in previous studies by our group [18,19] time-kill curves were generated in all cases of relapse to test the activity of ampicillin/gentamicin or ampicillin/ceftriaxone against the *E*. *faecalis* strains of patients whose initial treatment failed. Synergy was defined as decrease >2 logarithm in colony forming units (cfu)/mL with an antibiotic combination compared with each drug in monotherapy; Bactericidal activity: a decrease >3 log cfu/mL with the combination compared with each drug in monotherapy. Indifference: when none of the two previous occurred.

### Statistical analysis

Categorical variables are summarized as percentages. Continuous variables are summarized as mean and standard deviation. Categorical variables were compared using the chi-square test (or Fisher exact test when necessary). Continuous variables were compared using the Kruskal-Wallis test. Survival analysis was performed by Kaplan-Meier analysis and curves compared by the log-rank test. Given the limited sample size, logistic regression analysis was not performed. A 2-sided P<0.05 was considered to be statistically significant. The statistical analysis was performed using SPSS for Windows, Version 16.0 (SPSS Inc, Chicago, Illinois, USA).

## Results

### Basal characteristics of EFIE patients

During the study period, we diagnosed 92 episodes of enterococcal IE, 14 of which were excluded from the analysis (2 cases of *E*. *faecium* IE, 3 treated with combinations other than ampicillin/gentamicin or ampicillin/ceftriaxone, 2 because of early death –less than 72h from diagnosis-, and 7 with pacemaker-lead–associated IE). The baseline characteristics of the 78 patients included are shown in Table 1.

### Clinical characteristics and outcomes

**Fig 1:**
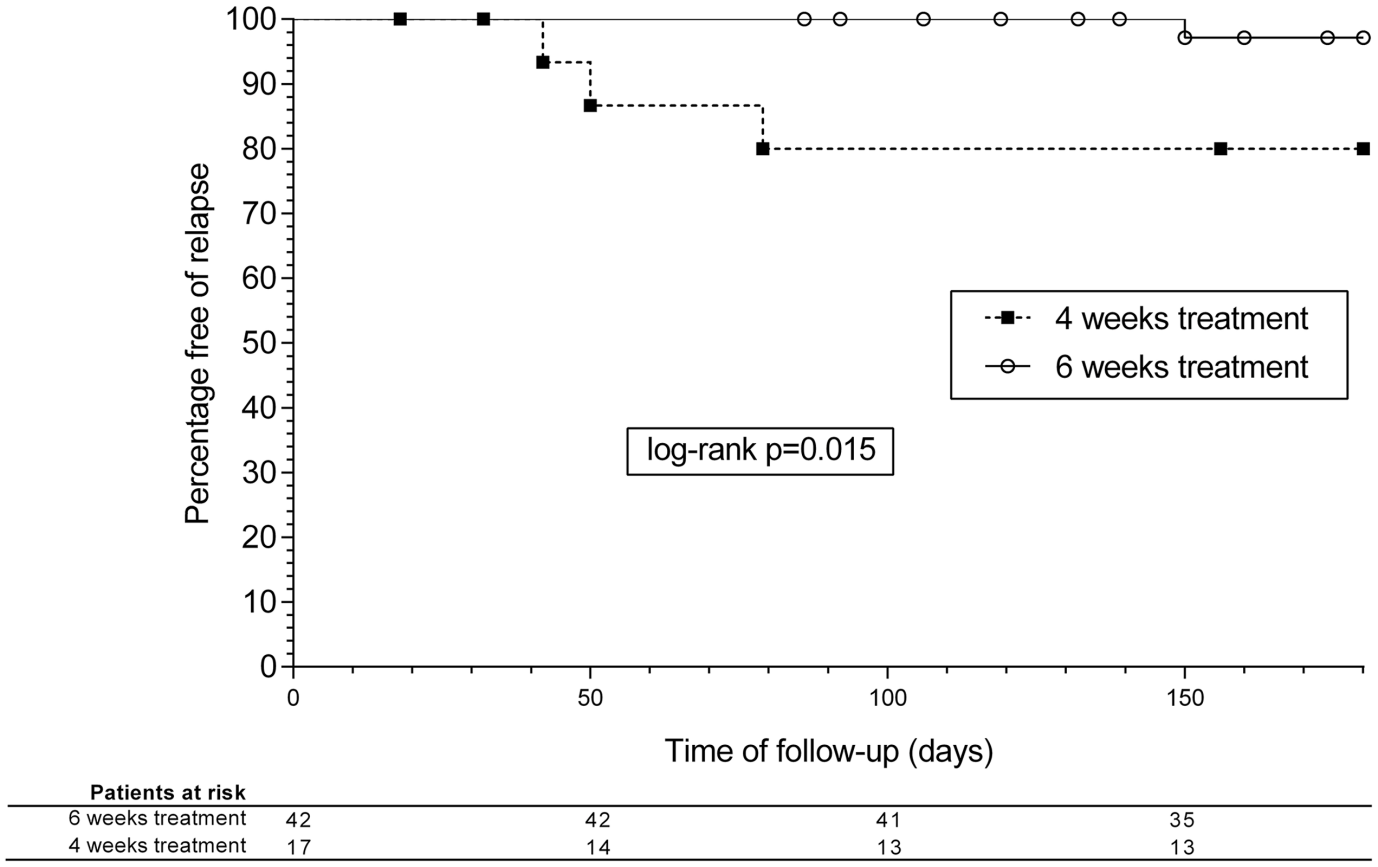
Kaplan-Meier survival analysis curves. One-year mortality according to the duration of treatment.

**Fig 2:**
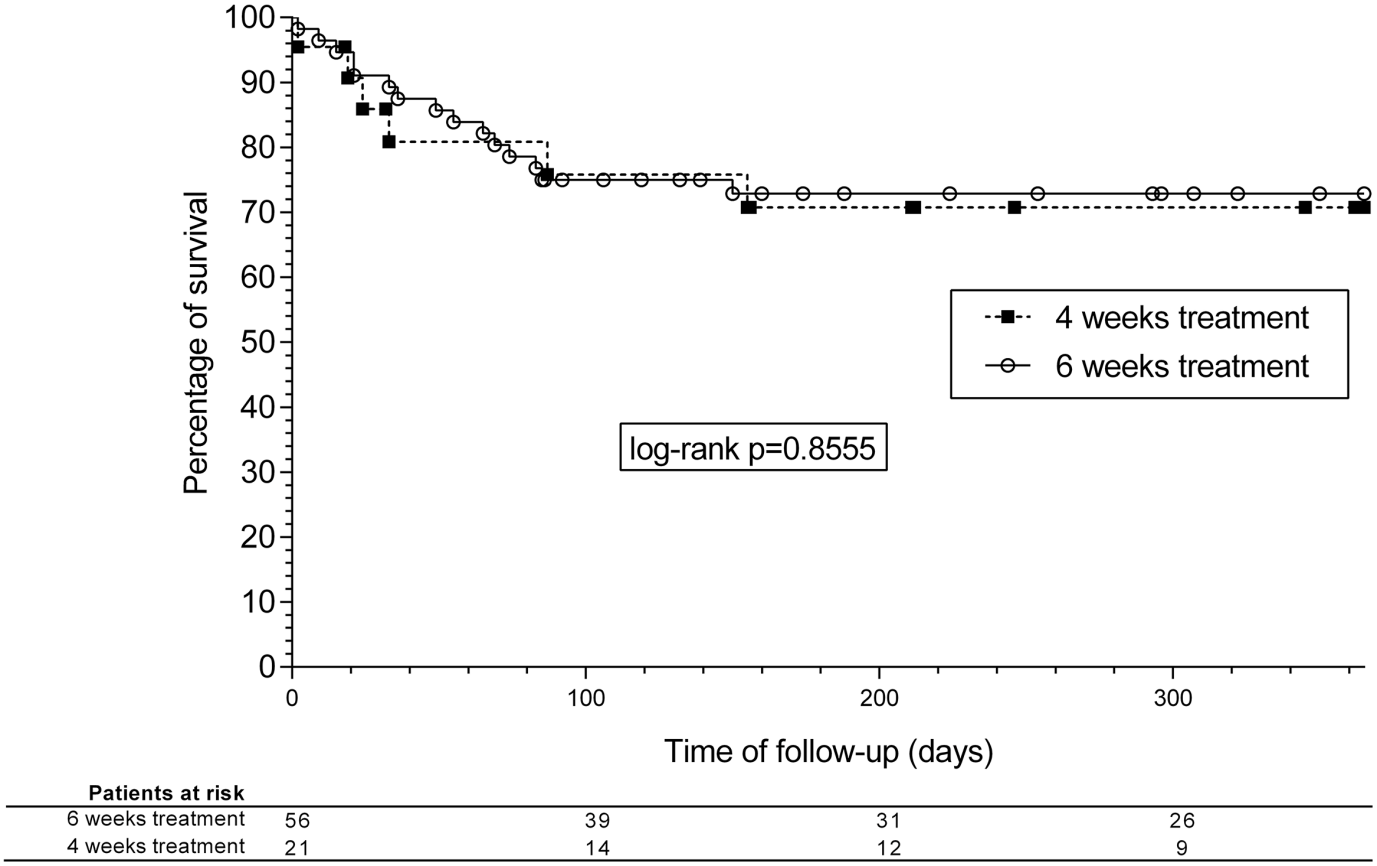
Kaplan-Meier survival analysis curves. Relapses at 180 days according to the duration of treatment.

The clinical picture of EFIE patients and the main outcomes can be seen in Table 2. Survival analysis curves for 1-year mortality are shown in Fig 1. There were no significant differences in mortality between the groups, although relapses were more frequent in patients receiving a 4-week course, in both ampicillin/gentamicin and ampicillin/ceftriaxone groups altogether and separately (3/18 [17%] and 1/41 [2%] in the 4- and 6-week groups, respectively; P = 0.045) (Fig 2). The overall rate of relapse was 5.1% (95%CI, 0.22-9.98%) and 6.8% considering only patients surviving the first admission due to EFIE. When analyzing only NVE episodes by treatment length (4 vs. 6 weeks), no significant differences were found in the rate of relapse (3 vs. 1 episode, P = 0.316).

### Clinical characteristics, treatment, and outcome of relapses

In brief, four relapses were diagnosed within the cohort. Three out of 4 (75%) relapses occurred in patients with advanced liver cirrhosis, and 3 (33%) of the 9 cirrhotic patients included in this series relapsed: 1/5 of those treated for 6 weeks and 2/4 of those treated for 4 weeks (Table 2). *E*. *faecalis* was not isolated from ascitic fluid in any cases. When the rate of relapses in non-cirrhotic patients treated for 4 weeks was analyzed, none of the 5 patients treated with ampicillin/gentamicin relapsed, and only 1 relapse (9%) was diagnosed in 11 patients treated with ampicillin/ceftriaxone.

#### Cases relapsing under treatment with ampicillin plus gentamicin

**Case 1 (4 weeks):** The patient was a 75-year-old woman with a history of dermatomyositis and severe lung involvement who had acute aortic native valve EFIE with HLSR in 2003 and was treated with 4 weeks of ampicillin/gentamicin (data published elsewhere) [18]. The relapse occurred 46 days after completing treatment; the patient presented with fever and positive blood cultures with the same strain of *E*. *faecalis*. No perivalvular complications, heart failure, or embolic events developed. She restarted ampicillin/gentamicin at the same dose, but 20 days later she developed renal failure and switched to ampicillin/ceftriaxone. Treatment was maintained for 22 days (total treatment time 6 weeks), and renal function recovered at the end of admission. She was alive and well after 1 year.

**Case 2 (6 weeks):** The patient was a 53-year-old man with Child B alcoholic cirrhosis who had been diagnosed with aortic native valve EFIE without HLAR in 2008. He had been treated with 6 weeks of ampicillin/gentamicin because of a periannular abscess. The patient was not initially considered a candidate for surgery owing to the high surgical risk. After a relapse 4 months after completing treatment, he was transferred to our center to be evaluated for surgery, since he had developed massive aortic insufficiency and an aorto-ventricular fistula. He developed renal failure after 3 weeks of treatment with ampicillin/gentamicin; therefore, therapy was switched to ampicillin/ceftriaxone. The patient completed 6 weeks of therapy with negative blood cultures and no fever. He underwent cardiac surgery, but died 20 days later from postsurgical multiorgan failure.

#### Cases relapsing under treatment with ampicillin plus ceftriaxone

**Case 3 (4 weeks):** The patient was a 65-year-old man with Child C liver cirrhosis who had been diagnosed with aortic NVIE and HLR to both aminoglycosides in 2009. He was treated initially with ampicillin/ceftriaxone for 4 weeks. He experienced a first relapse 3 weeks after completing treatment, and treatment with ampicillin/ceftriaxone was maintained for 6 weeks. Twenty-four hours after the end of treatment, he again presented with fever and positive blood cultures. Treatment was started with daptomycin 10 mg/kg and ampicillin 2 g/4 h, although ampicillin was withdrawn 5 days later owing to lack of synergy *in vitro* according to the time-kill curves. Ten days after completing 6 weeks of daptomycin, he developed fever, and blood cultures were positive for the same strain of *E*. *faecalis*; therefore, ampicillin monotherapy was started. The patient died from heart failure a few days later owing to worsening of initial valve function and fluid overload.

**Case 4 (4 weeks):** The patient was a 51-year-old man who was diagnosed with Child B HCV cirrhosis and native aortic valve non-HLAR EFIE in 2010 (with severe aortic insufficiency but not heart failure) and was initially treated with ampicillin/ceftriaxone for 4 weeks. He was not considered a candidate for surgery owing to liver disease and pancytopenia. He was readmitted 6 weeks after completing treatment with fever, and blood cultures again yielded *E*. *faecalis*. As time-kill curves demonstrated a lack of synergy for ampicillin/ceftriaxone, the patient was treated with ampicillin for 6 weeks and gentamicin for 4 weeks. He remained alive after 2 years of follow-up.

The time-kill curves showing the *in vitro* susceptibility of the strains causing the relapse in each case are depicted in Table 3. The combination of ampicillin plus gentamicin was synergistic and reached a bactericidal effect in all the strains, except against the isolate from case 3, which presented HLR against both aminoglycosides. The double beta-lactam combination was synergistic in all cases with the exception of case 3 and showed a bactericidal effect against case 1 and case 4. The combination of daptomycin plus ampicillin only increased the *in vitro* activity against the isolate from case 3, which was the one treated with that combination as rescue therapy. However, synergy and bactericidal activity were not reached either in this case. No antagonism was observed with any combination against any strain.

**Table 3:** Susceptibility patterns and time-kill curves with ampicillin plus gentamicin, ceftriaxone, or daptomycin against the strains causing the 4 relapses.

*Relapses*	Case 1		Case 2		Case 3		Case 4	
*Treatment*	A+G		A+G		A+C		A+C	
*Duration*	4 wk		6 wk		4 wk		4 wk	
**Susceptibility profiles of the strains (MIC/MBC)**^+^								
AMP	1/>128		1/>128		2/>128		1/>128	
GEN	8/16		64/128		>512/>512 (HLAR)		8/16	
CRO	32/512		8/16		>128/>128		32/512	
DAP	1/16		2/8		1/8		1/8	
**Time-kill Curves**								
	4 h*	24 h*	4 h*	24 h*	4 h*	24 h*	4 h*	24 h*
**AMP+GEN**								
Control	+1.1	+1	+0.8	+0.8	+0.9	+0.7	+0.9	+0.2
AMP	-0.4	-1.3	-0.3	-0.4	-0.1	-0.1	-0.6	-0.9
GEN	-1.7	-0.8	+0.4	+0.8	+0.9	+0.6	+0.7	+0.8
AMP+GEN	-2.7	-3.6	-2.5	-3	0	-0.1	-2.3	-3.1
**AMP-CRO**								
Control	+1.3	+1.2	+1.2	+0.9	+0.9	+1	+1	+0.9
AMP	+0.2	-0.2	-0.5	-1.1	0	0	-0.2	-0.4
CRO	+0.6	0	-0.2	-0.6	+0.7	+1	+0.6	-0.3
AMP-CRO	-1.8	-3.5	-1.4	-2.8	-0.7	-1.9	-2.1	-4
**AMP-DAP**								
Control	+0.9	+1	+1	+0.9	+1.1	+0.6	+0.8	+0.7
AMP	-0.9	-1.7	-1	-2.4	+0.2	-0.3	-0.3	-2.5
DAP	-0.3	-0.1	-1	+0.5	-0.9	0	+0.3	+0.2
AMP+DAP	-0.8	-2.6	-1.1	-1.8	-1.1	-2.4	-1	-1.5

^+^ Expressed in μg/mL

* Change in log_10_ CFU/mL;

A+G: ampicillin plus gentamicin; A+C: ampicillin plus ceftriaxone; AMP: ampicillin; CRO: ceftriaxone; DAP: daptomycin; GEN: gentamicin; MIC: minimum inhibitory concentration; MBC: minimum bactericidal concentration; HLAR: high-level aminoglycoside resistance.

## Discussion

In the current study, the rate of relapses among patients receiving a 4-week course of antibiotic treatment (either ampicillin/gentamicin or ampicillin/ceftriaxone) was significantly higher than in patients receiving a 6-week treatment. This occurred despite patients was assigned to receive a 4-week course based on the recommendations of current international guidelines. This might call attention on the current use of a 4-week course for ampicillin/gentamicin, as recommended in guidelines, as well as to the caution and further data needed before recommending the use of a 4-week course of ampicillin/ceftriaxone, even in well-selected non-complicated cases.

AHA guidelines recommend longer courses (6 weeks) for patients presenting with prosthetic valve endocarditis or symptoms lasting >3 months, since Wilson *et al* found a 44% rate of relapse among patients treated for 4 weeks with a beta-lactam plus an aminoglycoside [8]. The study included 56 patients with EFIE (36 treated with penicillin and streptomycin and only 20 treated with penicillin and gentamicin owing to HLSR) and revealed that 28.6% had been symptomatic for more than 3 months at diagnosis and that 44% of those patients relapsed, although none of the patients with a shorter duration of symptoms relapsed. None of the 4 patients who relapsed in our cohort presented symptoms for more than 3 months in the initial episode. In fact, such a long duration of symptoms is unusual in current enterococcal endocarditis series [2,8,11–13,16,19].

Wilson *et al* also found that mitral valve involvement was significantly associated with a higher rate of relapse: 25% of patients with mitral IE relapsed, whereas none of those with aortic valve IE relapsed. In contrast, the 4 relapses in our study were in patients with aortic valve IE [8]. Consequently, although our findings confirm the intuition of Wilson et al—also assumed in the AHA guidelines—that a short, 4-week course of therapy in EFIE could lead to a higher rate of relapse owing to the demographic progression of EFIE during the last 30 years, the indications for long-term therapy used to date have become obsolete, and new criteria should probably be developed.

In this study, the overall efficacy of ampicillin/ceftriaxone was not inferior to that of ampicillin/gentamicin and was safer in terms of renal failure development leading to treatment discontinuation, which is consistent with previous studies [12] and preliminary data from our cohort [13]. In addition to an equal rate of relapses, the efficacy of ampicillin/ceftriaxone administered for 4 weeks is similar to that of ampicillin/gentamicin administered for 4 weeks, and only 1 of the 11 non-cirrhotic patients with native valve EFIE treated with ampicillin/ceftriaxone relapsed. Surgical and major complications were very similar in both groups, with the exception of the rate of discontinuation of antibiotic therapy, which was higher in patients taking ampicillin/gentamicin, mostly owing to renal failure. These findings suggest the convenience of a 2-week course of aminoglycosides instead of 4 weeks when using ampicillin/gentamicin, as proposed elsewhere [16, 19, 20].

Relapses occurred mostly in patients with advanced liver cirrhosis. In our series, 33% of cirrhotic patients relapsed: 2 patients received 4 weeks of ampicillin/ceftriaxone and 1 patient received 6 weeks of ampicillin/gentamicin (overall, relapses were observed in 50% of cirrhotic patients receiving 4 weeks of therapy and 20% of those who received 6 weeks). Fernandez-Guerrero *et al* found a 9.8% prevalence of liver cirrhosis in a cohort of 316 patients with IE. Compared with the remaining patients, cirrhotic patients more frequently had streptococcal and enterococcal IE and died more frequently after surgery when liver impairment was advanced [21]. No studies have prospectively analyzed the influence of liver cirrhosis in the occurrence of relapses of IE, although cirrhotic patients are especially vulnerable to invasive enterococcal infections, including EFIE, for a number of reasons: increased susceptibility to bacterial infections due to leukocyte dysfunction and phagocytic defects, changes in normal gastrointestinal flora due to lowered excretion of gastric acid, the higher risk of bacterial translocation through the intestinal wall [22], ascites as a potential long-term reservoir, and the rising incidence of *Enterococcus spp* in spontaneous bacterial peritonitis [23]. In addition, since cirrhotic patients have a high surgical risk, it is mandatory to avoid relapses and worsening of the clinical picture of IE, since this could leave surgery as the patient’s only recourse. Consequently, we advocate a 6-week regimen for all cirrhotic patients with previous or current liver decompensation. This issue should be reassessed in further studies.

Our study has several limitations. First, the retrospective design prevents for drawing firm conclusions in regard exclusively with the length of the treatment. Second, the study period is large. This could introduce bias because the heterogeneity of study population. Third, statistical power is limited owing to the small sample size. Fourth, in contrast with other studies, referral bias probably led us to include nonstandard EFIE patients. On the other hand, there are not published studies supporting the guidelines recommendation of the use of 4-week courses of treatment for uncomplicated native valve EFIE. This is the first study analyzing this subject; hence larger studies will be necessary to clarify the potential role of short-course regimens (for both ampicillin/gentamicin and ampicillin/ceftriaxone).

In conclusion, due to an increased rate of relapse in those patients treated for 4 weeks of ampicillin/gentamicin and ampicillin/ceftriaxone, the suitability of a short course of antibiotic treatment for uncomplicated EFIE and its indications should be readdressed. For now, ampicillin/ceftriaxone for 4 weeks cannot be recommended, and ampicillin/gentamicin for 4 weeks needs to be indicated carefully. These results might provide the rationale for a clinical trial in uncomplicated native valve EFIE.

## Appendix

Members of the Hospital Clínic Endocarditis Study Group, Hospital Clínic-IDIBAPS, University of Barcelona School of Medicine, Barcelona, Spain: Manel Almela, MD, Juan Ambrosioni, MD, PhD, Manuel Azqueta, MD, Marta Bodro, MD, PhD, Merce Brunet, MD, PhD, Pedro Castro, MD, PhD, Carlos Falces, MD, David Fuster, MD, PhD, Guillermina Fita, MD, Cristina Garcia-de-la-Maria, PhD, Javier Garcia-Gonzalez, MS, Jose M. Gatell, MD, PhD, Marta Hernandez-Meneses, MD, Jaume Llopis, MD, PhD, Francesc Marco, MD, PhD, José M. Miró, MD, PhD, Asuncion Moreno, MD, PhD, José Ortiz, MD, PhD, David Nicolas, MD, Salvador Ninot, MD, J. Carlos Paré, MD, PhD, Juan M Pericàs, MD, PhD, Eduard Quintana, MD, PhD, Jose Ramirez, MD, PhD, Irene Rovira MD, Elena Sandoval, MD, Marta Sitges, MD, PhD, Adrian Tellez, MD, José M. Tolosana, MD, PhD, Barbara Vidal, MD, PhD, Jordi Vila, MD, PhD.

## Supporting information

S1 Dataset Dataset with demographic, microbiological, clinical, and therapeutic information on the 78 cases included in the study. (SAV)
