# Correction: Outcome of *Enterococcus faecalis* infective endocarditis according to the length of antibiotic therapy: Preliminary data from a cohort of 78 patients

**DOI:** 10.1371/journal.pone.0196317

**Published:** 2018-04-19

**Authors:** Juan M. Pericàs, Carlos Cervera, Asunción Moreno, Cristina Garcia-de-la-Mària, Manel Almela, Carles Falces, Eduard Quintana, Bàrbara Vidal, Jaume Llopis, David Fuster, Carlos A. Mestres, Francesc Marco, Jose M. Miró, on behalf of the Hospital Clinic Endocarditis Study Group

The images for Figs 1 and 2 are incorrectly switched. The image that appears as Fig 1 should be Fig 2, and the image that appears as Fig 2 should be Fig 1. The figure captions appear in the correct order. Please see the corrected figures here.

**Fig 1:**
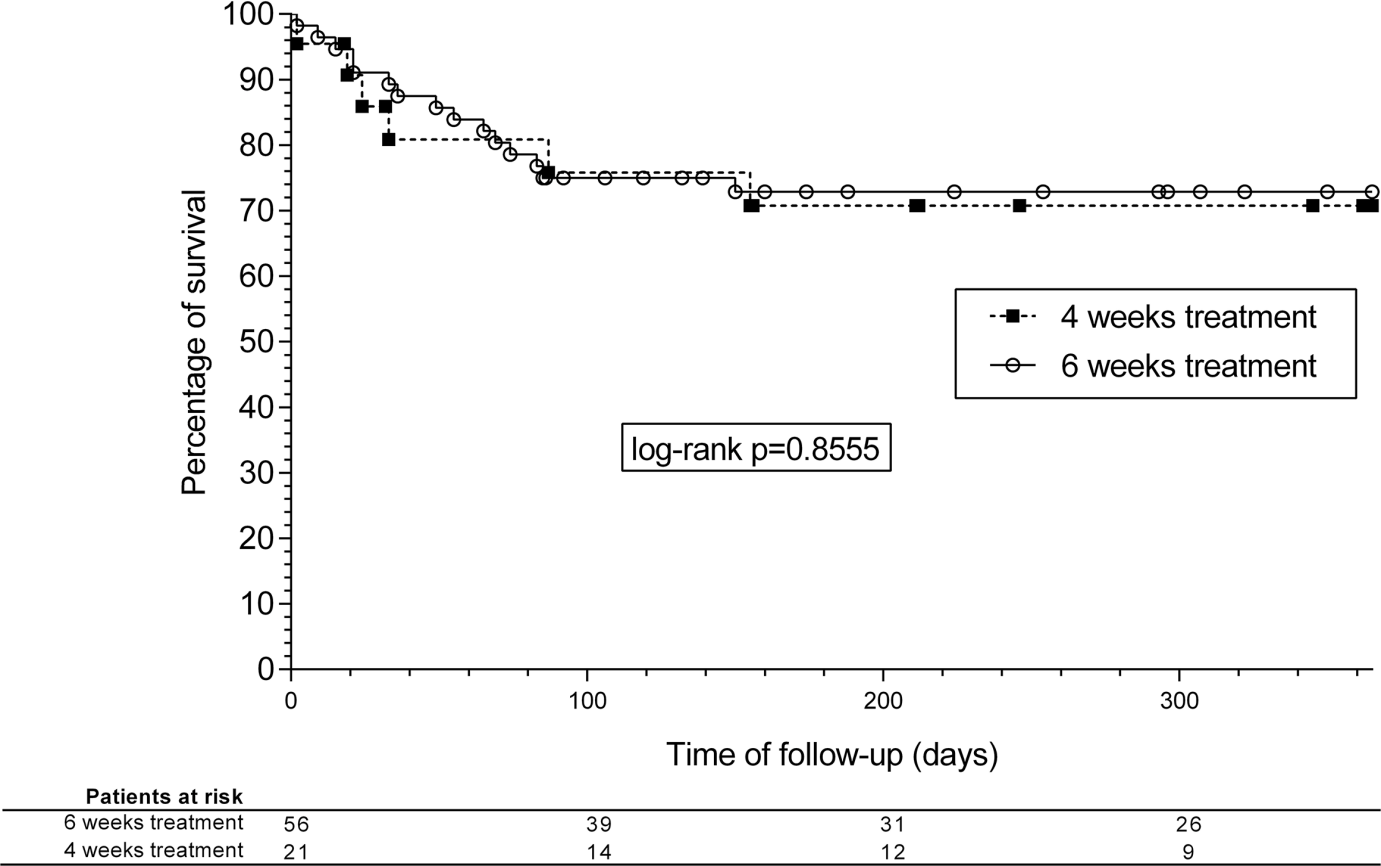
Kaplan-Meier survival analysis curves. One-year mortality according to the duration of treatment.

**Fig 2:**
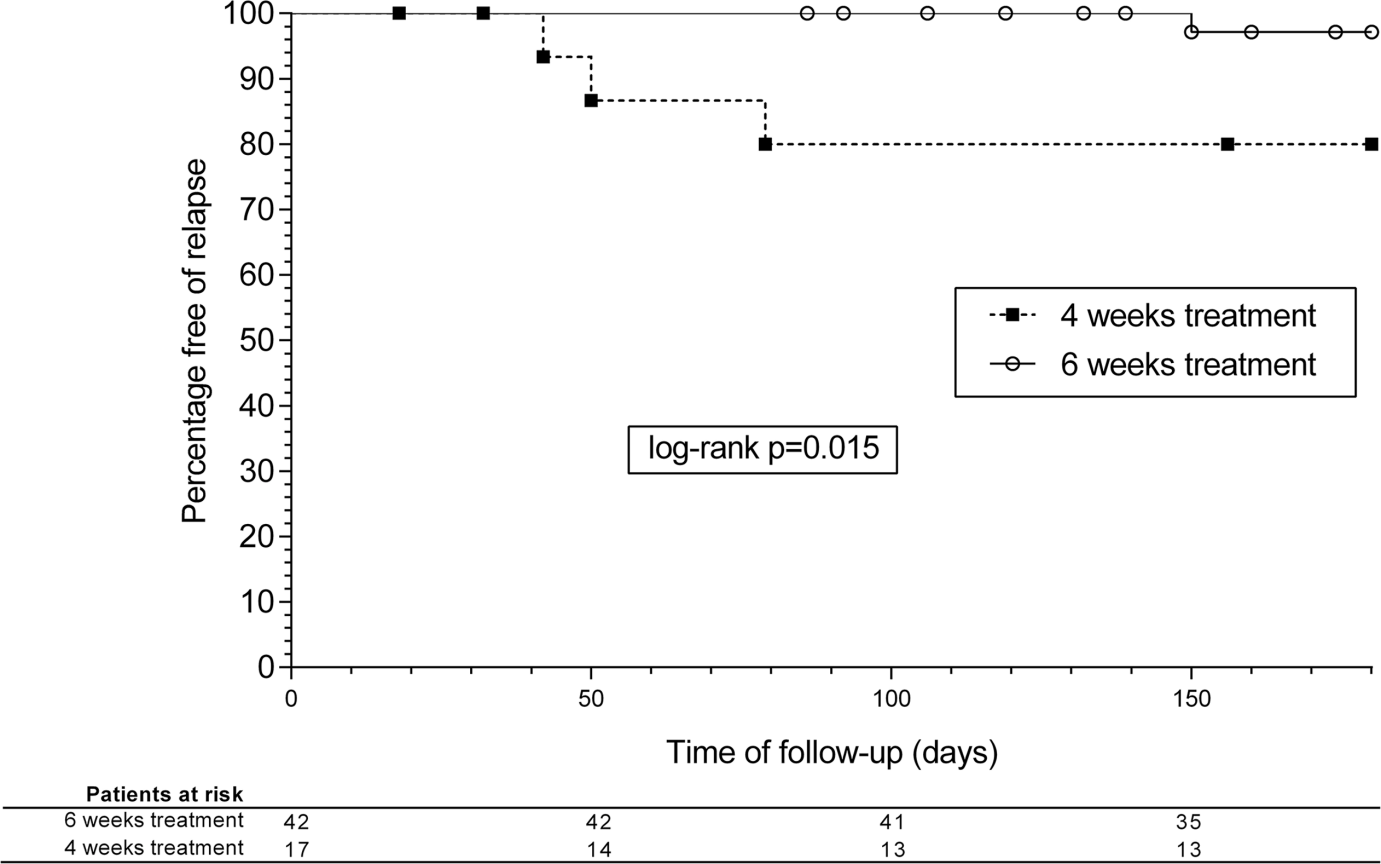
Kaplan-Meier survival analysis curves. Relapses at 180 days according to the duration of treatment.

## References

[1] Pericàs JM, Cervera C, Moreno A, Garcia-de-la-Mària C, Almela M, Falces C, et al (2018) Outcome of *Enterococcus faecalis* infective endocarditis according to the length of antibiotic therapy: Preliminary data from a cohort of 78 patients. PLoS ONE 13(2): e0192387 https://doi.org/10.1371/journal.pone.0192387 29462176 10.1371/journal.pone.0192387 PMC5819798

